# Efficacy and safety of Lemborexant in treating adult patients with insomnia in China: a single-center, retrospective observational study

**DOI:** 10.3389/fneur.2025.1495965

**Published:** 2025-03-14

**Authors:** Weiying Jian, Minyan Feng, Yifan Zhao, Jin Li

**Affiliations:** ^1^Department of Mental Health, Guangzhou United Family Hospital, Guangzhou, Guangdong, China; ^2^Department of Anesthesiology, Guangzhou United Family Hospital, Guangzhou, Guangdong, China; ^3^Department of Pharmacy, Guangzhou United Family Hospital, Guangzhou, Guangdong, China

**Keywords:** Lemborexant, insomnia, Chinese patients, the insomnia severity index, the patient health questionnaire-9, the general anxiety disorder-7

## Abstract

**Background:**

Except for one case report, there has been no published study of Lemborexant treatment for patients with insomnia in China. This study investigated efficacy and safety of Lemborexant in treating Chinese patients with insomnia.

**Methods:**

In this single-center, retrospective observational study, adult patients diagnosed with insomnia with an Insomnia Severity Index (ISI) score of ≥8 who were prescribed Lemborexant at Guangzhou United Family Hospital from January 2023 to July 2024 and who had ≥2 follow-up ISI assessment(s) were included. The primary outcome was change in the ISI total score from baseline after 4 weeks of Lemborexant treatment. Treatment-emergent adverse events (TEAEs) were collected.

**Results:**

Forty patients with a mean baseline ISI score of 17.0 ± 3.3 were included. The treatment continuation rate during the median 8-week (range: 2–20) follow-up was 90%. The ISI total score was reduced significantly from baseline after 4 weeks of treatment (−10.2 ± 3.0, *p* < 0.001), and was further reduced after 8 weeks of treatment (−12.7 ± 3.7, *p* < 0.001). Significant improvement in ISI total score at week 8 over week 4 was also observed. Both the Patient Health Questionnaire-9 and the General Anxiety Disorder-7 scores improved significantly after 4 weeks and 8 weeks of treatment. Thirty five (87.5%) patients were Lemborexant responders (ISI < 8). Age, combination therapy and Lemborexant 10 mg qn were independent factors associated with Lemborexant responders. One (2.5%) patient experienced mild dizziness. No patient discontinued the treatment due to TEAE(s).

**Conclusion:**

Lemborexant treatment was effective and safe in treating a wide variety of Chinese patients with different symptom(s) of insomnia.

## 1 Introduction

Insomnia is a common sleep disorder characterized by difficulty in initiating and/or maintaining sleep at least 3 nights per weeks for at least 3 months (1, 2). Insomnia interferes with daytime functioning, is detrimental to physical and mental health and is associated with lower quality of life and increased healthcare costs (3, 4). Insomnia has an estimated prevalence of 15.0% in China (5). Cognitive behavioral therapy (CBT) is considered a first-line treatment for insomnia and is effective in some patients (2). However, when CBT is ineffective or inaccessible, pharmacotherapy becomes necessary (2). Benzodiazepine (BZD) receptor (BZDR) agonists including both BZDs and non-BZDR agonist Z drugs are the most commonly prescribed insomnia medications (4). BZDR agonists are positive allosteric modulators of *γ*-aminobutyric acid type A (GABA-A) subunit α1 receptor and facilitate sleep by broadly inhibiting central nervous system activities (4). BZDR agonists are associated with next-morning residual effect, impaired memory and/or cognition, increased risk of accident, loss of efficacy over time, withdrawal symptoms/rebound insomnia upon discontinuation and risk of abuse and dependence (4–6). Therefore, their long-term use is not recommended (4, 6).

Lemborexant is a novel dual orexin receptor antagonist (DORA) approved for treating adults with insomnia in the United States, Canada, Japan, Australia and some other Asian countries (3). It is at present under review by the Center for Drug Evaluation (CDE) in China. Lemborexant binds competitively, reversibly and quickly to both orexin receptor types 1 and 2 (OX1R and OX2R), with higher affinity to OX2R (1, 2, 4). Therefore, unlike BZDs and Z drugs, Lemborexant reduces wakefulness and induces sleep by inhibiting orexin-mediated wake drive (3, 4, 7). Two crucial phase 3, randomized controlled trials (RCTs) examined the efficacy and safety of Lemborexant in treating adults with insomnia and found that Lemborexant 5 mg and 10 mg once every night (qn) provided both short-term and long-term benefits to sleep onset and sleep maintenance in adults with insomnia and were well tolerated (1, 2, 7). In addition, Lemborexant did not lead to rebound insomnia or withdrawal symptoms upon discontinuation, had little next-morning residual effect and did not affect cognition, driving or other aspects of daily functioning (4, 8).

Except for one case report (9), there has been no published study of using Lemborexant to treat adult patients with insomnia in China. We conducted a single-center, retrospective observational study to evaluate efficacy and safety of Lemborexant in treating Chinese adult patients with insomnia. Such a study could pave the way for future clinical trials and could also be useful to physicians in China who wish to consider Lemborexant as a treatment option for their patients with insomnia.

## 2 Materials and methods

### 2.1 Study design and patients

This is a single-center, retrospective observational study. Patients who visited the Department of Mental Health, Guangzhou United Family Hospital from January 16, 2023 to July 16 2024 were eligible for the study. Inclusion criteria: (1) Adult patients diagnosed with insomnia by our psychiatrists according to the Diagnostic and Statistical Manual of Mental Disorders, Fifth Edition (DSM-5) and had a baseline Insomnia Severity Index (ISI) score of ≥8; (2) were prescribed Lemborexant for the first time as regular treatment between January 16, 2023 to July 162,024; and (3) had at least 2 follow-up visits and at least 2 post-baseline ISI assessment(s). Our hospital’s electronic medical records were reviewed to select patients who fit the inclusion criteria. All of the included patients gave written informed consent to participate in the study and all data were anonymized. This study was approved by the Institutional Review Board of Guangzhou United Family Hospital (Approval number: GZHMJ-2024-07-01; Date: July 16, 2024).

Electronic medical records of the enrolled patients were reviewed and the following data were collected: gender, age, ethnicity, height, body weight, body mass index (BMI), level of education, marriage status, living arrangement, employment status, months since symptom onset and since initial insomnia diagnosis, reason(s) for the visit when Lemborexant was prescribed, any psychiatric condition(s) affecting sleep, other past/present comorbidities and concomitant medication(s). In addition, baseline and follow-up ISI scores, the Patient Health Questionnaire-9 (PHQ-9) score and the General Anxiety Disorder-7 (GAD-7) score, the Clinical Global Impressions Scale-Improvement (CGI-I) score, regimen adjustment and treatment emergent adverse events (TEAEs) were also collected.

### 2.2 Efficacy outcome measures

Primary outcome measure was change in the ISI total score from baseline after 4 weeks of Lemborexant treatment. The self-reported ISI includes seven items: (1) Difficulty in sleep onset; (2) difficulty in sleep maintenance; (3) early morning awakening; (4) dissatisfaction with sleep; (5) interference with daytime functioning; (6) noticeability of the sleep difficulties by others; and (7) distress due to sleep difficulties. Each of the seven items was scored by patients on a 0 (no problem) to 4 (very serious problem) scale, and the ISI total score was the sum of the 7 scores. An ISI total score of 0–7, 8–14, 15–21, and 22–28 indicates no clinically significant insomnia, subthreshold insomnia, moderate insomnia and severe insomnia, respectively (3).

Secondary outcome included: (1) Changes in the scores of the 7 items of the ISI from baseline after 4 weeks of Lemborexant treatment; (2) change in the ISI total score from baseline after 8 weeks of treatment; (3) changes in the PHQ-9 and the GAD-7 scores from baseline after 4 weeks and 8 weeks of treatment; (4) difference between the week 4 and week 8 ISI, PHQ-9 and GAD-7 scores; (5) the proportion of Lemborexant responders (ISI < 8), and the proportions of patients with 50 and 75% improvement (reduction) in their ISI scores; (6) the proportions of patients whose symptoms improved, remained the same or worsened according to their clinician-rated CGI-I scores after 4 weeks and 8 weeks of treatment. A CGI-I score of 1–3, 4 and 5–7 indicates improved, unchanged and worsened symptoms, respectively (10).

In addition, the Kaplan–Meier curve was plotted for non-responders (ISI ≥ 8) to assess the patients’ response to Lemborexant treatment over time.

Subgroup analysis of change in the ISI total score from baseline after 4 weeks of Lemborexant treatment were performed to compare efficacy of Lemborexant for patients with psychiatric comorbidities vs. those without psychiatric comorbidities. In addition, subgroup analyses of changes in the ISI total score, the PHQ-9 and the GAD-7 scores from baseline after 4 weeks of treatment based on concomitant hypnotics and/or antidepressant(s) and on Lemborexant dosage were also performed.

Finally, independent factors associated with Lemborexant responders (ISI < 8) were identified.

### 2.3 Safety

Treatment emergent adverse events (TEAEs) and their severity were recorded.

### 2.4 Statistical analysis

All statistical analyses in the study were performed with R version 4.3.1 (The R Foundation for Statistical Computing, Vienna, Austria). Descriptive statistics was used. Normality of data distribution was assessed with the Shapiro–Wilk Test. Categorical variables were expressed as numbers and percentages [*N* (%)], while continuous variables with normal and non-normal distributions were expressed as means ± standard deviations (x̄ ± s) and median (Interquartile range), respectively. Independent samples *t*-test was used to compare the week 4 vs. week 8 ISI, PHQ-9 and GAD-7 scores, while paired *t*-test or repeated-measures ANOVA was used to assess other changes in the scores. The Kaplan–Meier curve was plotted for non-responders (ISI ≥ 8) to assess the patients’ response to Lemborexant treatment over time. Finally, stepwise variable selection for Cox regression was utilized to identify independent factors associated with Lemborexant responders (ISI < 8). Statistical significance was accepted with a two-tailed *p* < 0.05.

## 3 Results

### 3.1 Demographics and baseline characteristics

**Figure 1:**
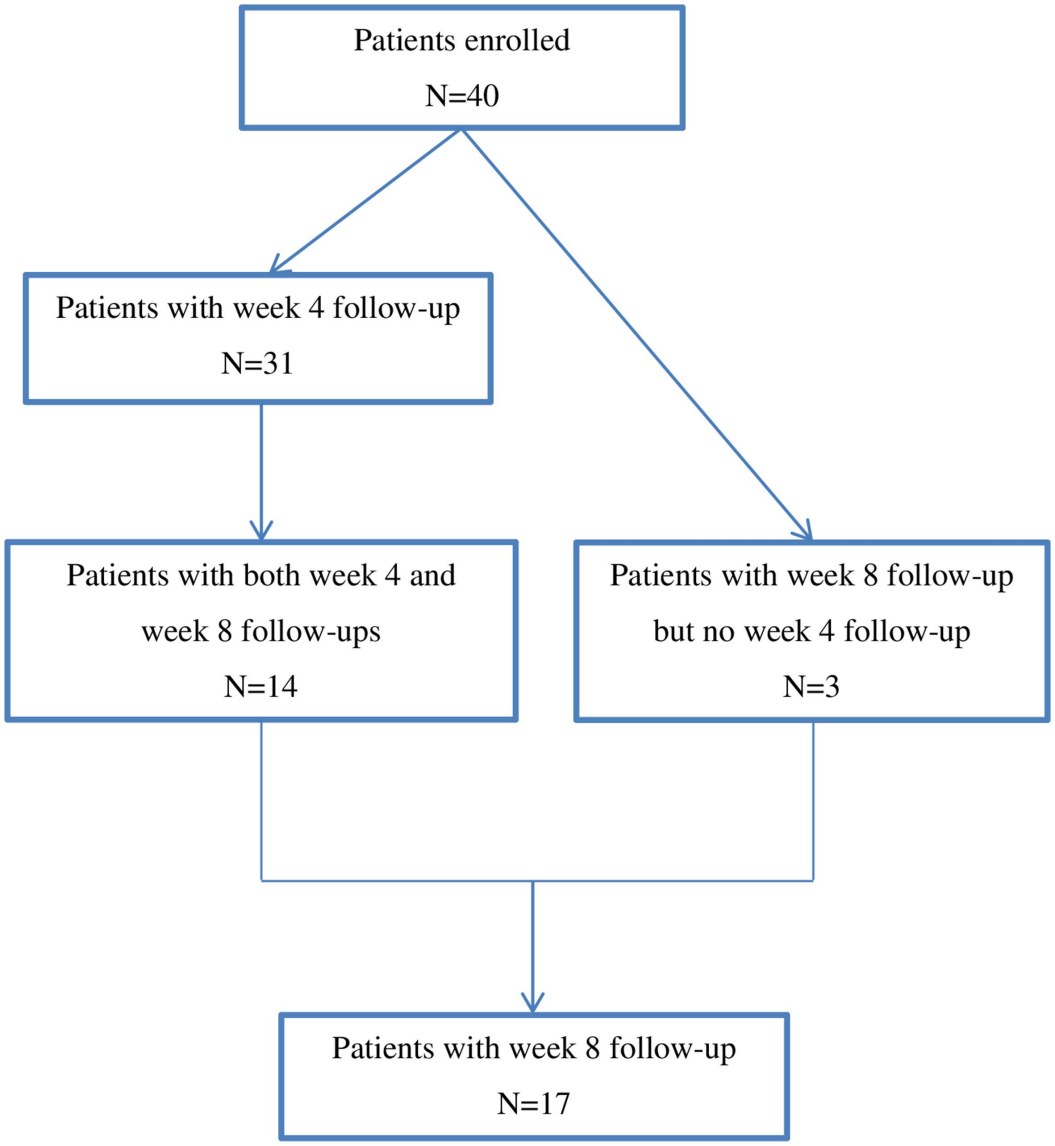
Study flow chart.

The study flow chart was depicted in Figure 1. A total of 40 patients were included in the study. Demographics and baseline characteristics of the patients were described in Table 1. The 40 patients included 27 females and 13 males with a mean age of 44.7 ± 12.3 years. At the time of being prescribed Lemborexant, 45.6 ± 63.7 months had passed since their initial insomnia diagnosis. Among them, 22 (55.0%) had psychiatric comorbidities that affected sleep. Twelve (30.0%) patients did not take concomitant medication(s), while 17 (42.5%) and 20 (50.0%) patients had been taking concomitant antidepressant(s) and hypnotics, respectively. Their mean baseline ISI score was 17.0 ± 3.3 (Table 1).

**Table 1:** Demographics and baseline characteristics.

	All patients (*N* = 40)	Patients with week 4 follow-up (*N* = 31)	Patients with week 8 follow-up (*N* = 17)
Female, *n* (%)	27 (67.5%)	22 (71.0%)	13 (76.5%)
Age, years, x̄ ± *s*	44.7 ± 12.3	43.7 ± 11.2	46.5 ± 10.7
20–39 years, *n* (%)	16 (40%)	13 (41.9%)	5 (29.4%)
40–59 years, *n* (%)	17 (42.5%)	13 (41.9%)	9 (52.9%)
≥ 60 years, *n* (%)	7 (17.5%)	5 (16.1%)	3 (17.6%)
Height, cm, x̄ ± s	164.5 ± 7.6	164.7 ± 7.3	162.2 ± 8.0
Body weight, kg, x̄ ± s	61.0 ± 11.1	60.2 ± 10.0	61.6 ± 12.7
BMI, kg/m^2^, x̄ ± s	22.5 ± 3.4	22.2 ± 3.4	23.3 ± 4.0
Marriage status, *n* (%)			
Unmarried	5 (12.5%)	4 (12.9%)	0 (0.0%)
Married	35 (87.5%)	27 (87.1%)	17 (100.0%)
Employment status, *n* (%)			
Full-time	22 (55.0%)	18 (58.1%)	8 (47.1%)
Retired	6 (15.0%)	5 (16.1%)	2 (11.8%)
Unemployed	8 (20.0%)	4 (12.9%)	3 (17.6%)
Part-time	1 (2.5%)	2 (6.5%)	2 (11.8%)
Freelance	3 (7.5%)	2 (6.5%)	2 (11.8%)
Months since symptom onset, x̄ ± s	76.4 ± 82.5	74.6 ± 81.4	68.4 ± 56.6
Missing data, *n* (%)	1 (2.5%)	1 (3.2%)	0 (0.0)
Months since initial insomnia diagnosis, x̄ ± s	45.6 ± 63.7	52.3 ± 69.7	39.1 ± 52.9
Reason for the visit when Lemborexant was prescribed, *n* (%)			
First visit	2 (5.0%)	1 (3.2%)	1 (5.9%)
Recurrence of insomnia	4 (10.0%)	3 (9.7%)	0 (0.0)
Aggravated symptom(s)	11 (27.5%)	7 (22.6%)	5 (29.4%)
Maintenance treatment	10 (25.0%)	8 (25.8%)	4 (23.5)
No change in symptoms	13 (32.5%)	12 (38.7%)	7 (41.2%)
Psychiatric condition(s) affecting sleep, *n* (%)			
Yes	22 (55.0%)	18 (58.1%)	10 (58.8%)
No	18 (45.0%)	13 (41.9%)	7 (41.2%)
Other past / present comorbidities, *n* (%)			
Yes	7 (17.5%)	6 (19.4%)	3 (17.6%)
No	33 (82.5%)	25 (80.6%)	14 (82.4%)
Concomitant medication^a^			
None	12 (30.0%)	9 (29.0%)	5 (29.4%)
Concomitant antidepressant(s)	17 (42.5%)	13 (41.9%)	5 (29.4%)
Other hypnotics	20 (50.0%)	16 (51.6%)	11 (64.7%)
ISI, x̄ ± s	17.0 ± 3.3	16.6 ± 3.1	17.1 ± 2.9

^a^Some patients took both antidepressant(s) and BZRAs. x̄±s, means ± standard deviations; BMI, body mass index; BZDR, benzodiazepine receptor; ISI, Insomnia Severity Index.

### 3.2 Rate of treatment continuation and doses

The 40 patients had a median follow-up of 8 weeks (range: 2–20 weeks). Among them, four (10%) patients discontinued the treatment because their symptoms improved substantially and their ISI scores dropped below 8. None of them experienced rebound insomnia or withdrawal syndrome upon discontinuation. All of the remaining 36 patients continued the treatment on their latest visits, and the rate of treatment continuation was 90%.

The percentage of patients taking Lemborexant 5 mg qn and 10 mg qn were 97.5% (38) and 2.5% (2) at baseline, respectively, while 93.5% (29), and 6.5% (2) at week 4, respectively. At the week 8 visit, all of the 17 patients were prescribed Lemborexant 5 mg qn.

### 3.3 Changes of the ISI scores and the proportions of Lemborexant responders (ISI < 8)

After 4 weeks of Lemborexant treatment, the ISI total score was reduced significantly from baseline (−10.2 ± 3.0, *p* < 0.001), and was further reduced after 8 weeks of treatment (−12.7 ± 3.7, *p* < 0.001). Significant improvement in the week 8 ISI total over the week 4 ISI score was also observed (−2.03 ± 1.02, *p* = 0.048) (Table 2).

**Table 2:** Changes in the ISI total score, PHQ-9 and GAD-7 scores from baseline after 4 weeks and 8 weeks of Lemborexant treatment.

	Week 4 vs. baseline (*N* = 31)				
	Baseline	Week 4	Change from baseline	*t*-value	*p*-value
ISI	16.6 ± 3.1	6.4 ± 3.1	−10.2 ± 3.0	18.7	<0.001
PHQ-9	7.4 ± 3.7	3.7 ± 2.6	−3.6 ± 3.5	5.7	<0.001
GAD-7	6.3 ± 4.8	3.3 ± 3.3	−3 ± 3.5	5.1	<0.001

	Week 8 vs. baseline (*N* = 17)				
	Baseline	Week 8	Change from baseline	*t*-value	*p*-value
ISI	17.1 ± 2.9	4.4 ± 3.3	−12.7 ± 3.7	11.8	<0.001
PHQ-9	7.2 ± 3.1	2.7 ± 2.5	−4.5 ± 3.6	5.0	<0.001
GAD-7	7.2 ± 4.4	3.2 ± 2.8	−4 ± 4.2	3.6	0.003

	Week 8 vs. week 4 (*N* = 14)			
	Week 8	Change from Week 4	*t*-value	*p*-value
ISI	4.4 ± 3.4	−2.03 ± 1.02	2.06	0.048
PHQ-9	2.7 ± 2.5	−1.42 ± 0.44	1.36	0.182
GAD-7	3.2 ± 2.8	−0.2 ± 0.29	0.06	0.952

ISI, insomnia severity index; PHQ-9, Patient Health Questionnaire-9; GAD-7, General Anxiety Disorder-7.

Furthermore, all of the 3 sleep parameters (sleep initiation, maintenance and early morning awakening) as well as all of the 4 daytime functioning parameters of the ISI were significantly reduced after 4 weeks of treatment (*p* < 0.001) (Table 3).

**Table 3:** Changes in the scores of the 7 items of the ISI from baseline after 4 weeks of Lemborexant treatment.

*N* = 31	Baseline	Week 4	Change from baseline	*t*-value	*p*-value
Difficulty in sleep onset	3 ± 1	1.2 ± 0.9	−1.8 ± 0.9	11.4	<0.001
Difficulty in sleep maintenance	2.8 ± 0.9	1.2 ± 0.6	−1.6 ± 1	9.2	<0.001
Early morning awakening	1.7 ± 1.3	0.4 ± 0.7	−1.3 ± 1.2	5.9	<0.001
Sleep dissatisfaction	2.7 ± 0.8	1.2 ± 0.5	−1.5 ± 0.9	10.1	<0.001
Interference with daytime functioning	2.2 ± 0.5	0.9 ± 0.6	−1.2 ± 0.7	10.2	<0.001
Noticeability of the sleep problems by others	2.1 ± 0.6	0.9 ± 0.7	−1.2 ± 0.7	9.4	<0.001
Distress caused by the sleep problems	2.1 ± 0.6	0.7 ± 0.7	−1.4 ± 0.7	11.8	<0.001

ISI, insomnia severity index.

**Figure 2:**
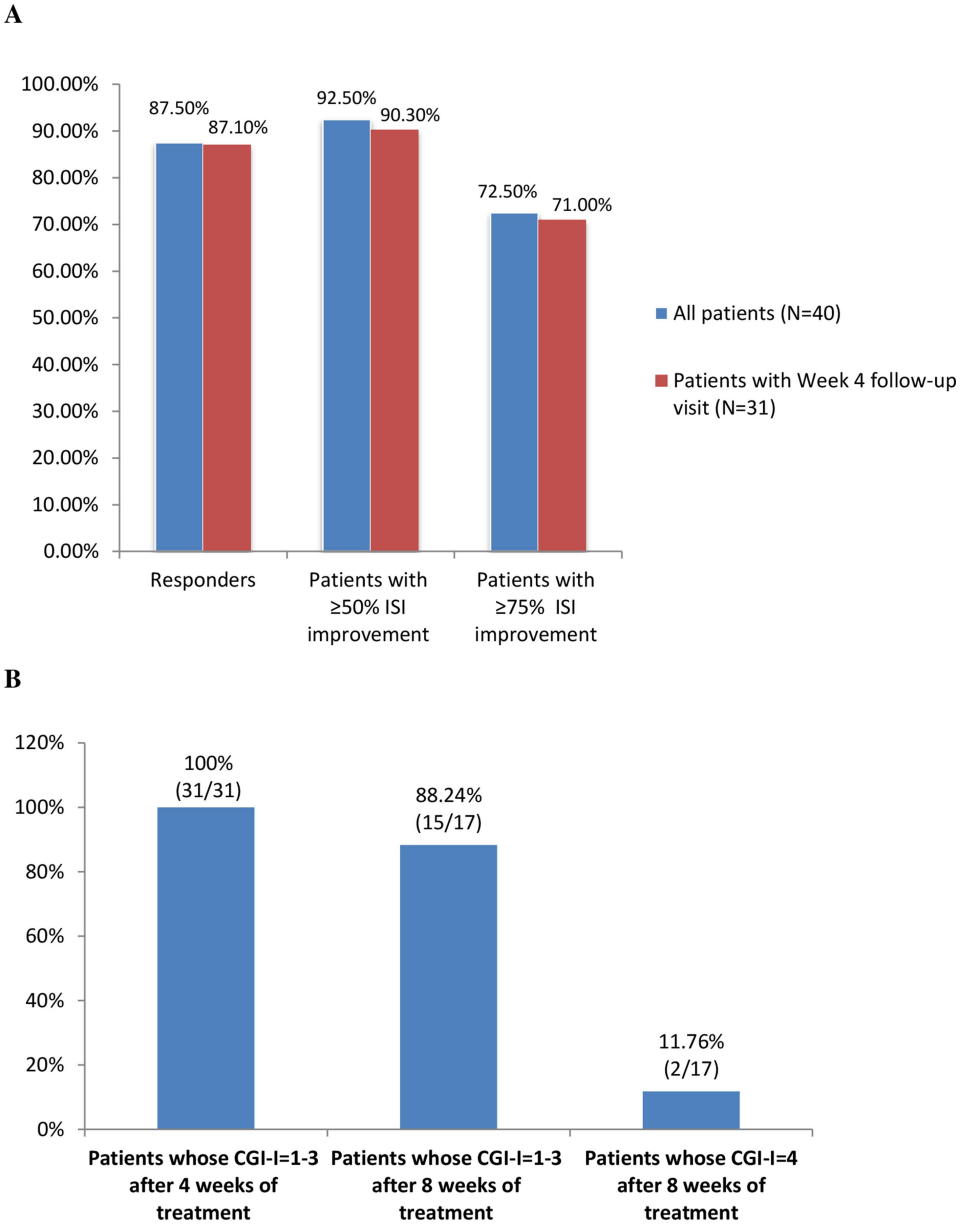
**(A)** The proportions of Lemborexant responders (ISI < 8) and patients with ≥50% and ≥ 75% improvements in their ISI scores. **(B)** The proportion of patients whose condition improved (CGI-I = 1–3) and whose condition remained the same (CGI-I = 4) after 4 weeks and 8 weeks of Lemborexant treatment. Abbreviations: ISI, Insomnia Severity Index; CGI-I, the Clinical Global Impressions Scale-Improvement.

Among the 40 included patients, 35 (87.5%) were Lemborexant responders, while 37 (92.5%) and 29 (72.5%) patients had 50 and 75% improvement in their ISI total scores, respectively. Similar results were observed for the 31 patients with week 4 follow-up (Figure 2A).

According to the clinician-rated CGI-I, all of the 31 (100%) patient with week 4 follow-up had improvement (CGI-I = 1–3) after 4 weeks of treatment. Meanwhile, among the 17 patients with week 8 follow-ups, 15 (88.24%) and 2 (11.76%) patients showed improvement and remained the same (CGI-I = 4), respectively (Figure 2B).

**Figure 3:**
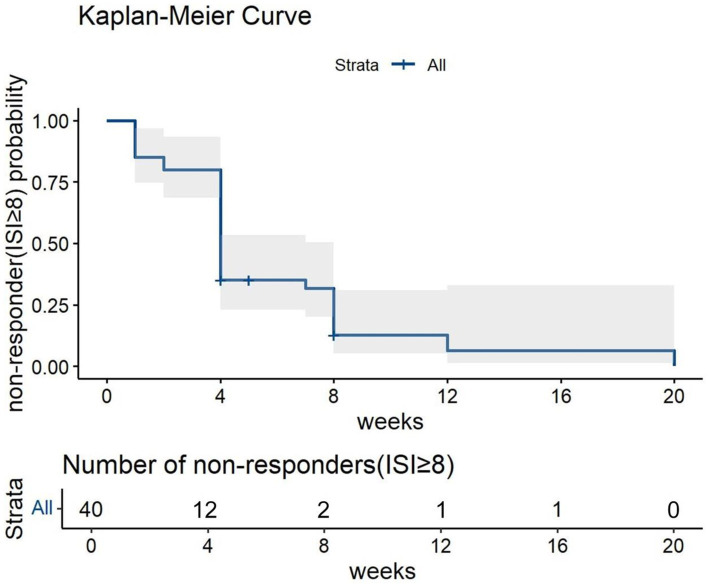
The Kaplan–Meier Curve for non-responders to Lemborexant treatment over the follow-up period.

The Kaplan–Meier curve for non-responders (ISI ≥ 8) showed decreasing proportion of non-responders over the follow-up period. In addition, the proportion of non-responder drastically decreased after 4 weeks of treatment (Figure 3).

### 3.4 Improvement of psychiatric comorbidities with Lemborexant treatment

Comparable reductions in the ISI total scores from baseline after 4 weeks of Lemborexant treatment were observed for patients with psychiatric comorbidities (*N* = 18) vs. those without psychiatric comorbidities (*N* = 13) (−10.3 ± 2.9 vs. −10.1 ± 3.3, *p* = 0.978).

Significant reductions in the PHQ-9 score from baseline were observed after both 4 weeks and 8 weeks of Lemborexant treatment (−3.6 ± 3.5, *p* < 0.001; −4.5 ± 3.6, *p* < 0.001, respectively). Meanwhile, the GAD-7 score also decreased significantly after 4 weeks and 8 weeks of treatment (−3 ± 3.5, *p* < 0.001; −4 ± 4.2, *p* = 0.003, respectively). The effect of Lemborexant treatment on the PHQ-9 and GAD-7 scores after 4 weeks of treatment were maintained over the 8 weeks of treatment (−1.42 ± 0.44, *p* = 0.182; −0.2 ± 0.29, *p* = 0.952, respectively) (Table 2).

### 3.5 Subgroup analyses of changes in the ISI total score, the PHQ-9 and the GAD-7 scores from baseline after 4 weeks of Lemborexant treatment

**Figure 4:**
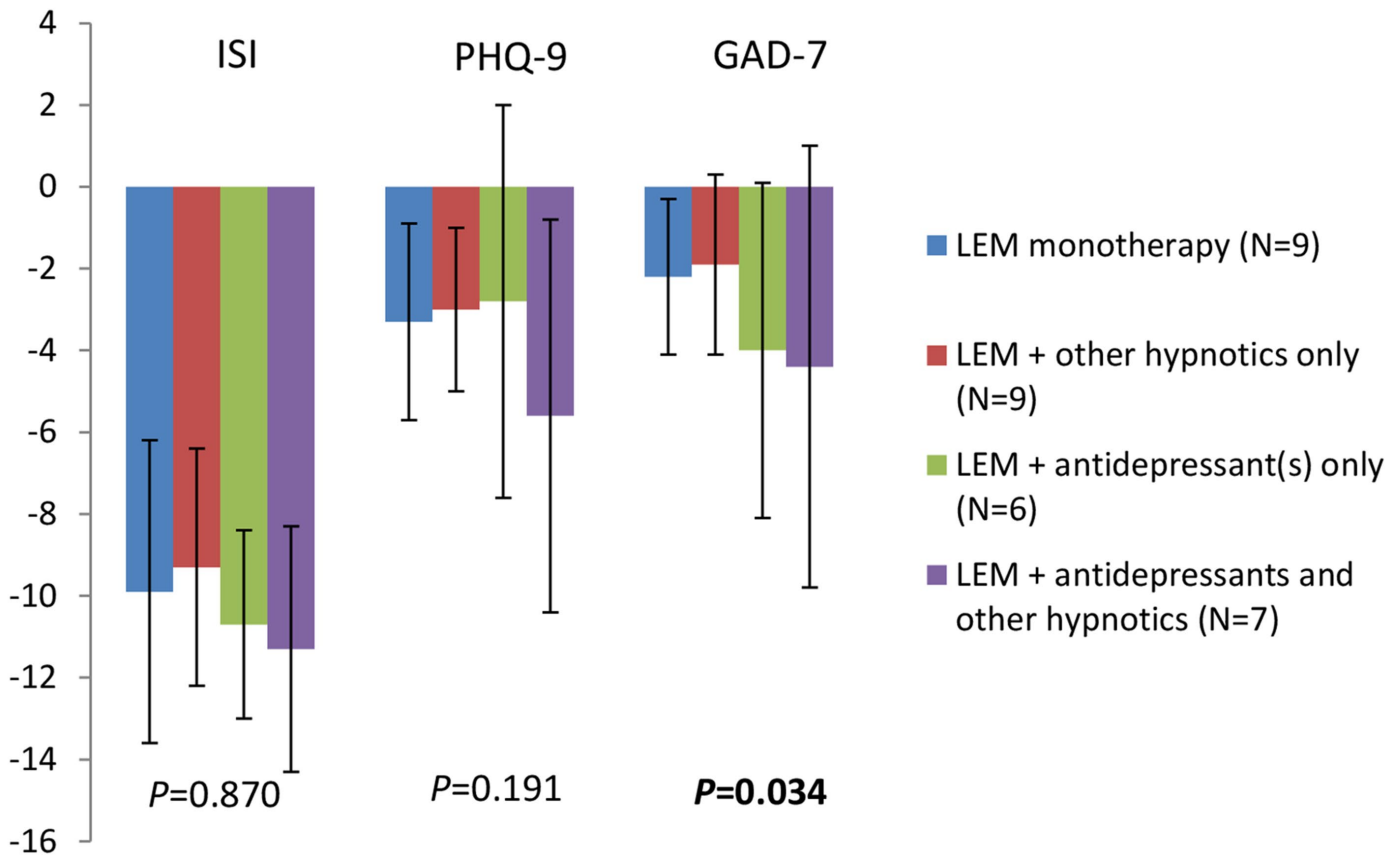
Changes in the ISI total score, the PHQ-9 and the GAD-7 scores from baseline after 4 weeks of treatment for patients receiving Lemborexant monotherapy, Lemborexant + other hypnotics only, Lemborexant + antidepressant(s) only, and Lemborexant + antidepressant(s) and other hypnotics. LEM, Lemborexant; ISI, insomnia severity index; PHQ-9, Patient Health Questionnaire-9; GAD-7, General Anxiety Disorder-7.

After 4 week of treatment, among patients receiving Lemborexant monotherapy (*N* = 9), Lemborexant + other hypnotics only (*N* = 9), Lemborexant + antidepressant(s) only (*N* = 6) and Lemborexant + antidepressant(s) and other hypnotics (*N* = 7), there was no significant difference in the ISI total score reductions (−9.9 ± 3.7, −9.3 ± 2.9, −10.7 ± 2.3, and − 11.3 ± 3.0, respectively, *p* = 0.870) and in the PHQ-9 score reductions (−3.3 ± 2.4, −3.0 ± 2.0, −2.8 ± 4.8, and − 5.6 ± 4.8, respectively, *p* = 0.191), although the Lemborexant + antidepressant(s) and other hypnotics group had numerically greater decrease in the PHQ-9 score (Figure 4 and Supplementary Table S1). On the other hand, reductions in the GAD-7 scores from baseline were significantly different among these four subgroups of patients (−2.2 ± 1.9, −1.9 ± 2.2, −4.0 ± 4.1, and − 4.4 ± 5.4, respectively, *p* = 0.034), wherein patients receiving Lemborexant + antidepressant(s) only and patients receiving Lemborexant + antidepressant(s) and other hypnotics had greater GAD-7 score reduction than patients in the other two subgroups (Figure 4 and Supplementary Table S1).

In addition, after 4 weeks of treatment, between patients receiving Lemborexant 5 mg qn (*N* = 29) and 10 mg qn (*N* = 2), there was no significant difference in reductions in the ISI total scores (−9.9 ± 2.8 vs. −14.5 ± 3.5, *p* = 0.901), the PHQ-9 (−3.6 ± 3.5 vs. −4.5 ± 3.5, *p* = 0.486) and the GAD-7 scores (−3.1 ± 3.5 vs. −1.0 ± 1.4, *p* = 0.147) from baseline, although patients taking Lemborexant 10 mg qn had numerically greater decrease in the ISI total score (Supplementary Table S2).

### 3.6 Independent factors associated with Lemborexant responders (ISI < 8)

Stepwise variable selection for Cox regression revealed that age, combination therapy and Lemborexant 10 mg qn were independent factors associated with Lemborexant responders. The likelihood of a patient being a responder decreased with increasing age (HR [95%CI]: 0.955 [0.912, 1.000], *p* = 0.048). On the other hand, patients receiving Lemborexant combination therapy were more likely to be responders than those receiving monotherapy (HR [95%CI]: 3.546 [1.023, 12.294], *p* = 0.046), and patients receiving Lemborexant 10 mg qn were more likely to be responders than those receiving 5 mg qn (HR [95%CI]: 13.683 [2.094, 89.394], *p* = 0.006) (Table 4).

**Table 4:** Demographics and baseline characteristics that affected whether a patient was a responder (ISI < 8) or not.

	Cox regression			Stepwise selection		
	HR (95% CI)	z	Pr(>|z|)	HR (95% CI)	z	Pr(>|z|)
Presence of psychiatric comorbidities	0.867 (0.299, 2.518)	−0.262	0.794	–	–	–
Moderate insomnia vs. subthreshold insomnia^a^	0.724 (0.157, 3.33)	−0.415	0.678	–	–	–
Severe insomnia vs. subthreshold insomnia	0.214 (0.019, 2.42)	−1.245	0.213	–	–	–
Age	0.973 (0.912, 1.039)	−0.815	0.415	0.955 (0.912, 1.000)	−1.979	**0.048**
Married vs. unmarried	1.051 (0.147, 7.527)	0.049	0.961			
Past use of insomnia medication(s) vs. no past use	0.126 (0.018, 0.883)	−2.086	**0.037**	0.232 (0.045, 1.192)	−1.750	0.080
BMI	0.945 (0.842, 1.06)	−0.971	0.332	–	–	–
Unchanged /maintenance treatment vs. first visit	3.119 (0.354, 27.488)	1.024	0.306	–	–	–
Follow-up visit vs. first visit	8.901 (0.658, 120.414)	1.645	0.100	–	–	–
Worsened insomnia vs. first visit	4.69 (0.535, 41.124)	1.395	0.163	–	–	–
Employed vs. unemployed	0.462 (0.108, 1.977)	−1.041	0.298	0.528 (0.209, 1.337)	−1.346	0.178
Retired vs. unemployed	2.026 (0.213, 19.229)	0.615	0.539	3.495 (0.811, 15.065)	1.679	0.093
Combination therapy vs. monotherapy	5.658 (1.225, 26.142)	2.219	**0.027**	3.546 (1.023, 12.294)	1.995	**0.046**
10 mg qn vs. 5 mg qn	30.979 (1.85, 518.84)	2.388	**0.017**	13.683 (2.094, 89.394)	2.732	**0.006**

^aAn ISI score of 22–28 indicated severe insomnia, a score of 15–21 indicated with moderate insomnia, and a score of 6–14 indicated subthreshold insomnia. ISI, Insomnia Severity Index; BMI, body mass index; qn, once every night.Bold values indicate statistical significance at the p < 0.05 level.^

In addition, baseline severity of insomnia (subthreshold, moderate or severe) did not affect whether a patient was a Lemborexant responder, neither did whether the visit when Lemborexant was prescribed was a first visit, follow-up visit, unchanged/maintenance treatment visit, or a visit due to worsened insomnia (Table 4).

### 3.7 Safety

One (2.5%) patients receiving Lemborexant 5 mg qn - zolpidem tartrate 5 mg qn combination treatment reported mild dizziness and the patient continued the treatment. None of the patients discontinued the treatment due to TEAE(s).

## 4 Discussion

In the 1-month, global, phase 3 RCT SUNRISE 1, compared with zolpidem tartrate extended-release (ZOL-ER) and placebo, Lemborexant treatment significantly improved sleep onset and maintenance including during the second part of the night according to both the objective polysomnography and the subjective sleep diary in patients aged ≥55 years with insomnia (2). In addition, the 12-month, global, phase 3 RCT SUNRISE 2 compared Lemborexant with placebo in patients aged ≥18 years with insomnia and found that Lemborexant 5 mg qn and 10 mg qn led to significantly greater improvement in sleep onset and maintenance than placebo and that the effectiveness of Lemborexant treatment was maintained over 12 months (1, 7). Findings from these studies demonstrated that Lemborexant was effective in promoting sleep onset and maintenance and that its effectiveness was long-term and thus could be used to treat patients with chronic insomnia (1, 2, 4, 7).

Our single-center, retrospective observational study was designed to evaluate efficacy and safety of Lemborexant in treating Chinese adult patients with insomnia. Lemborexant treatment significantly improved ISI in Chinese patients with insomnia in our study. Their ISI total score decreased significantly after 4 weeks and 8 weeks of treatment. Furthermore, significantly more pronounced week 8 decrease in ISI score over week 4 was observed, suggesting decreasing insomnia severity and sustained efficacy of Lemborexant treatment. Our results were consistent with prior studies (2, 3, 11). In the SUNRISE 1 study, significantly improved ISI total score was observed in patients ≥55 years old with insomnia after 1 month of Lemborexant treatment (−7.8 and − 7.9 for Lemborexant 5 mg qn and 10 mg qn, respectively) (2), while in the SUNRISE 2 study, the respective decrease in the ISI total scores in patients with insomnia treated with Lemborexant 5 mg qn and 10 mg qn were 7.1 and 7.2 after 1 month of treatment, respectively, and 10.8 and 10.2 after 3 months of treatment, respectively (3). The significantly greater improvement in the week 8 ISI score over week 4 in our study was also consistent with the Kaplan–Meier curve depicting decreasing proportion of Lemborexant non-responders over the follow-up period.

All of the 7 individual scores of the ISI decreased significantly after 4 weeks of treatment in our study, suggesting that Lemborexant treatment improved sleep initiation, maintenance and early morning awakening as well as daytime functioning. Importantly, the SUNRISE 1 study found that Lemborexant also improved sleep maintenance during the second half of the night compared with ZOL-ER (2, 12).

The rate of Lemborexant responders (ISI < 8) after 4 weeks in our study was 87.5%, higher than the 45.1% in Ozone et al. (13) and the 31.0% in Arnold et al. (14). The fact that patients in our study were younger (mean age: 44.7 years old) than patients in those two studies might play a role in the difference, as our study showed that the likelihood of a patients being a Lemborexant responder decreased with increasing age. In addition, our study was a retrospective, observational study, while the 2 previous studies were both controlled interventional studies (13, 14), difference in study designs could contribute to the difference in the rates of Lemborexant responders.

In our study, 22 (55%) patients had psychiatric comorbidities. Lemborexant treatment improved mood in our patients as well, as evidenced by the significantly decreased PHQ-9 and GAD-7 scores after both 4 weeks and 8 weeks of treatment. In addition, our subgroup analysis revealed that Lemborexant treatment was as effective in patient with psychiatric comorbidities as in those without psychiatric comorbidities. These findings were consistent with prior studies (8, 9, 15). As there are bidirectional relationships between insomnia and psychiatric conditions such as depression and anxiety (16–18), our observations that Lemborexant treatment was effective in treating insomnia in patients with psychiatric comorbidities and in alleviating symptoms of depression and anxiety are important.

Age, combination therapy and Lemborexant 10 mg qn were independent factors associated with Lemborexant responders in our study. The likelihood of a patient being a responder decreased with increasing age, although this negative effect of increasing age was small. An aged-based subgroup analysis revealed comparable proportions of Lemborexant responders among patients aged 20–39 years, 40–59 years, and ≥ 60 years in our study (Supplementary Figure S1), also suggesting a small effect of increasing age on a patient’s response to Lemborexant. Consistent with our finding, Lalovic et al. (19), an exposure-response analysis from the SUNRISE 1 and SUNRISE 2 studies, demonstrated that although age was a statistically significant covariate for subjective outcome measures, it was not therapeutically relevant.

Patients receiving Lemborexant combination therapy were more likely to be responders than those receiving monotherapy in our study. On the other hand, patients receiving Lemborexant monotherapy or combined with other hypnotics and/or antidrepressant(s) had comparable ISI total score reductions in our study. In addition, Mishima et al. (15) found that patients receiving add-on treatment were less likely to improve compared with patients receiving Lemborexant monotherapy. Whether and how combination treatment affect the efficacy of Lemborexant needs further investigation.

Studies have found that Lemborexant treatment has a good safety and tolerability profile and that headache and somnolence are the mostly commonly reported TEAEs (2, 3, 12). In addition, dizziness, sleep paralysis, nightmares, hallucinations, cataplexy and suicide ideation/attempt have also been reported (2, 3, 12, 16, 20, 21). According to a prospectively, post-marketing observational study, common TEAEs included somnolence (7.65%), nightmare (1.76%), abnormal dreams (0.59%) and sleep paralysis (0.20%) (15). Our study had a very low incidence of TEAEs (2.5%) and the only TEAE was mild dizziness. None of the patients in our study discontinued the treatment due to TEAEs. No other TEAE was reported in our study.

As previously reported (4, 8), none of the four patients who discontinued the treatment because their sleep improved substantially experienced any rebound insomnia or withdrawal symptoms after they stopped taking Lemborexant. Also consistent with previous studies (22, 23), patients in our study had a very high rate of treatment continuation suggestive of good efficacy, safety and tolerability of Lemborexant treatment.

This study has several limitations. First, it has a modest sample size and not all of the included patients had week 4 follow-up visits. In addition, most patients received Lemborexant 5 mg qn and only two patients received 10 mg qn. Further, there were only seven patients aged ≥60 years in the study. Therefore, our study may not have the statistical power adequate for some subgroup analyses such as dose-based efficacy analyses. For the same reason, whether age truly affects a patient’s response to Lemborexant treatment needs further investigations. Second, most of the patients in the study switched to Lemborexant treatment or received Lemborexant as an add-on therapy, and only one patient was naïve to hypnotics-sedatives before being prescribed Lemborexant. Third, it is a retrospective study which means that it is possible that clinicians missed potential TEAEs, which might be the reason why the incidence of TEAEs in our study was lower than previous studies (2, 3). Last, this study mainly studied efficacy and safety of 4-week and 8-week Lemborexant treatment, while its long-term efficacy and safety were not assessed. Long-term studies with a larger sample size are needed to confirm our findings. On the other hand, this study has its own strength. First, except for a case report (9), there has been no published study of Lemborexant treatment for insomnia in China, this study could potentially pave the way for future studies and provide some guidance to clinicians who wish to use Lemborexant as a treatment for their patients. Second, a real-world observational study like ours reflects daily clinical practice in China and could help to supplement and/or extrapolate data from RCTs. Third, although subjective or objective measures such as sleep diaries and polysomnography were not used in our study, our study employed both patient-reported ISI and physician-rated CGI-I to more accurately assess efficacy of Lemborexant.

## 5 Conclusion

In conclusion, Lemborexant was effective and safe in treating a wide variety of patients with different symptom(s) of insomnia in China and it was effective regardless of baseline severity of insomnia and whether the patients were naïve to hypnotics-sedatives, had worsened insomnia or seeking maintenance treatment when they were prescribed Lemborexant.

## Data Availability

The raw data supporting the conclusions of this article will be made available by the authors, without undue reservation.
