# Supplementary material for: Efficacy and safety of Lemborexant in treating adult patients with insomnia in China: a single-center, retrospective observational study
Source: Front Neurol. 2025 Mar 14;16:1495965. doi: 10.3389/fneur.2025.1495965 (PMC11949819; doi:10.3389/fneur.2025.1495965)
Supplement: Supplementary file 1 [file Data_Sheet_1.PDF]

SUPPLEMENTARY TABLE S1 Changes in the ISI total score, the PHQ-9 and the GAD-7 scores from baseline after 4 weeks of treatment for patients receiving LEM monotherapy, LEM + other hypnotics only, LEM + antidepressant(s) only, and LEM + antidepressant(s) and other hypnotics

		**Patients (N)**	**Baseline**	**Week 4**	**Change from baseline**	***p*-value**
**ISI**	LEM monotherapy	9	16.3±3.8	6.4±2.4	-9.9±3.7	0.870
	LEM + other hypnotics only	9	16.8±3.1	7.4±2.6	-9.3±2.9	
	LEM + antidepressant(s) only	6	15.8±2.6	5.2±0.8	-10.7±2.3	
	LEM + antidepressant(s) and other hypnotics	7	17.3±3.0	6.0±5.2	-11.3±3.0	
**PHQ-9**	LEM monotherapy	9	6.9±3.7	3.6±2.1	-3.3±2.4	0.191
	LEM + other hypnotics only	9	6.0±2.5	3.0±1.7	-3.0±2.0	
	LEM + antidepressant(s) only	6	8.0±3.6	5.2±3.3	-2.8±4.8	
	LEM + antidepressant(s) and other hypnotics	7	9.3±5.0	3.7±3.4	-5.6±4.8	
**GAD-7**	LEM monotherapy	9	4.7±3.7	2.4±2.7	-2.2±1.9	**0.034**
	LEM + other hypnotics only	9	4.4±4.0	2.6±2.4	-1.9±2.2	
	LEM + antidepressant(s) only	6	7.8±5.0	3.8±1.3	-4.0±4.1	
	LEM + antidepressant(s) and other hypnotics	7	9.3±5.9	4.9±5.6	-4.4±5.4	

Abbreviations: ISI, insomnia severity index; PHQ-9, Patient Health Questionnaire-9; GAD-7, General Anxiety Disorder-7; LEM, Lemborexant

SUPPLEMENTARY TABLE S2 Changes in the ISI total score, the PHQ-9 and the GAD-7 scores from baseline after 4 weeks of treatment for patients receiving Lemborexant 5mg qn and 10mg qn

		**Patients (N)**	**Baseline**	**Week 4**	**Change from baseline**	***p*-value**
ISI	5mg qn	29	16.4±3.0	6.6±3.1	-9.9±2.8	0.901
	10mg qn	2	18.5±4.9	4.0±1.4	-14.5±3.5	
PHQ-9	5mg qn	29	7.4±3.8	3.9±2.6	-3.6±3.5	0.486
	10mg qn	2	6.5±3.5	2.0±0.0	-4.5±3.5	
GAD-7	5mg qn	29	6.6±4.8	3.5±3.4	-3.1±3.5	0.147
	10mg qn	2	1.5±2.1	0.5±0.7	-1.0±1.4	

Abbreviations: ISI, insomnia severity index; PHQ-9, Patient Health Questionnaire-9; GAD-7, General Anxiety Disorder-7; qn, once every night
