# Supplementary figures and images for: Efficacy and safety of Lemborexant in treating adult patients with insomnia in China: a single-center, retrospective observational study
Source: Front Neurol. 2025 Mar 14;16:1495965. doi: 10.3389/fneur.2025.1495965 (PMC11949819; doi:10.3389/fneur.2025.1495965)

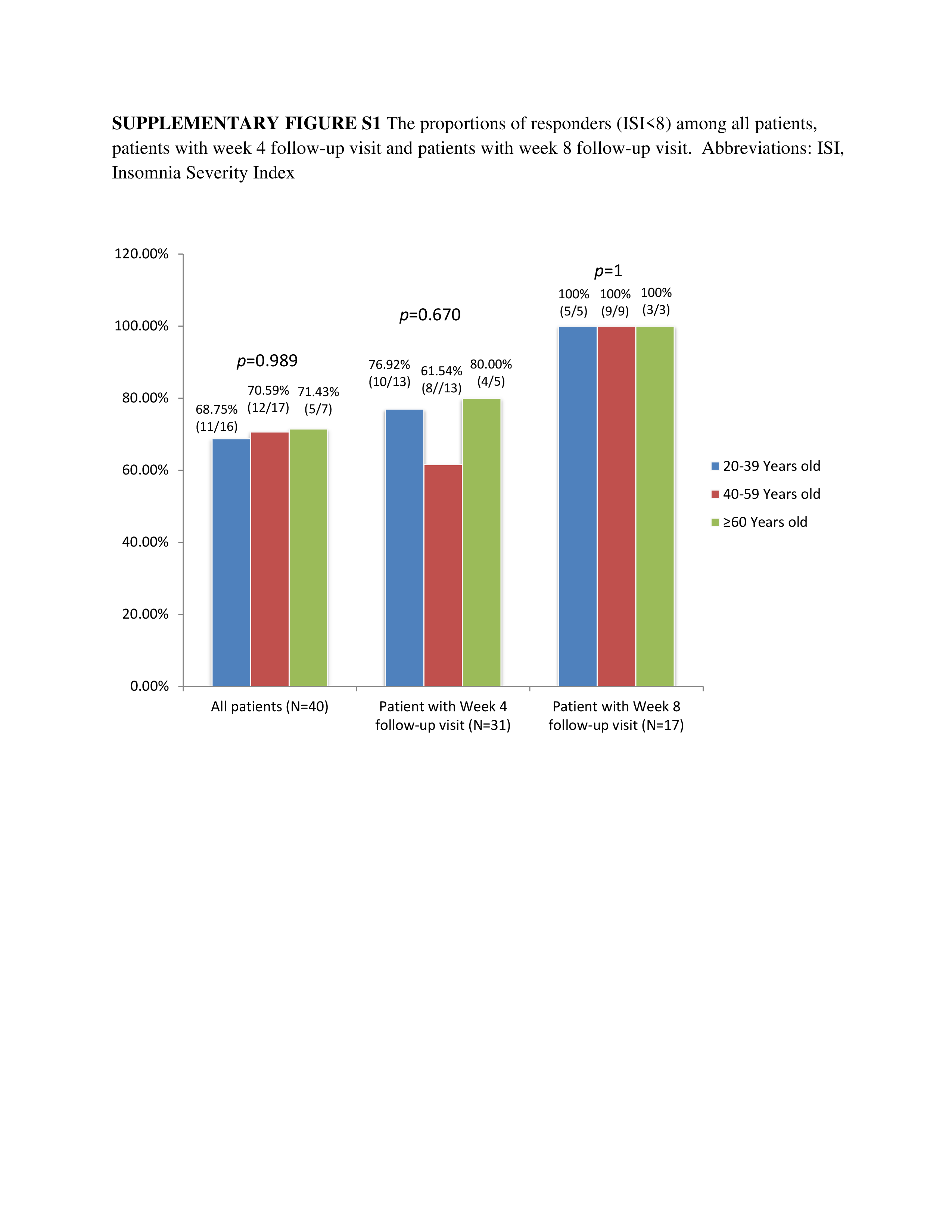

Supplement: Supplementary file 2 [file Image_1.tiff]
